# Evaluation of power wheelchair driving performance in simulator compared to driving in real-life situations: the SIMADAPT (simulator ADAPT) project—a pilot study

**DOI:** 10.1186/s12984-024-01354-5

**Published:** 2024-04-23

**Authors:** Bastien Fraudet, Emilie Leblong, Patrice Piette, Benoit Nicolas, Valérie Gouranton, Marie Babel, Louise Devigne, François Pasteau, Philippe Gallien

**Affiliations:** 1 LAB Saint Hélier, Pôle MPR St Hélier, 54 rue St Hélier, 35043 Rennes Cedex, France; 2 INSA Rennes, CNRS, Inria, University of Rennes, Rennes, France; 3 CNRS, IRISA, Inria, University of Rennes, Rennes, France

**Keywords:** Virtual reality, Driving simulator, Immersion robotics, Neurological disorders, Cybersickness, Power wheelchair

## Abstract

**Objective:**

The objective of this study was to evaluate users’ driving performances with a Power Wheelchair (PWC) driving simulator in comparison to the same driving task in real conditions with a standard power wheelchair.

**Methods:**

Three driving circuits of progressive difficulty levels (C1, C2, C3) that were elaborated to assess the driving performances with PWC in indoor situations, were used in this study. These circuits have been modeled in a 3D Virtual Environment to replicate the three driving task scenarios in Virtual Reality (VR). Users were asked to complete the three circuits with respect to two testing conditions during three successive sessions, i.e. in VR and on a real circuit (R). During each session, users completed the two conditions. Driving performances were evaluated using the number of collisions and time to complete the circuit. In addition, driving ability by Wheelchair Skill Test (WST) and mental load were assessed in both conditions. Cybersickness, user satisfaction and sense of presence were measured in VR. The conditions R and VR were randomized.

**Results:**

Thirty-one participants with neurological disorders and expert wheelchair drivers were included in the study. The driving performances between VR and R conditions were statistically different for the C3 circuit but were not statistically different for the two easiest circuits C1 and C2. The results of the WST was not statistically different in C1, C2 and C3. The mental load was higher in VR than in R condition. The general sense of presence was reported as acceptable (mean value of 4.6 out of 6) for all the participants, and the cybersickness was reported as acceptable (SSQ mean value of 4.25 on the three circuits in VR condition).

**Conclusion:**

Driving performances were statistically different in the most complicated circuit C3 with an increased number of collisions in VR, but were not statistically different for the two easiest circuits C1 and C2 in R and VR conditions. In addition, there were no significant adverse effects such as cybersickness. The results show the value of the simulator for driving training applications. Still, the mental load was higher in VR than in R condition, thus mitigating the potential for use with people with cognitive disorders. Further studies should be conducted to assess the quality of skill transfer for novice drivers from the simulator to the real world.

*Trial registration* Ethical approval n^∘^2019-A001306-51 from Comité de Protection des Personnes Sud Mediterranée IV. Trial registered the 19/11/2019 on ClinicalTrials.gov in ID: NCT04171973.

## Introduction

Power Wheelchairs (PWC) may be the only solution to maintain mobility and autonomy for some people with physical impairments. Several studies have underlined the benefits of using a PWC as it improves mobility and social participation while reducing the burden on caregivers [1], as well as the importance of moving for people with neurological impairments. For example, a study carried out by the Breizh Cerebral Palsy Network has shown the negative impact of limitations in moving around on the quality of life of people with cerebral palsy [2]. Independence in mobility was also identified as one of the main barriers to maintain societal participation in activities such as employment for people with traumatic brain injury (TBI) [3].

In this context, PWC driving confidence is therefore extremely important to guarantee the autonomy of people who need wheelchairs. One of the main barriers to autonomy through the access to mobility is that not everyone can access PWC driving as it can lead to accidents, in particular for people with visual or cognitive impairments. In fact, wheelchair safety reports show that 25$\%$ of wheelchair accidents are linked to the use of PWC [4]. Moreover, PWC driving is a difficult task which requires cognitive functions which can be impaired for people with neurological disorders. Indeed, up to 40$\%$ of people with TBI who use PWC regularly have problems with steering and 5$\%$ to 9$\%$ cannot steer at all in a clinical setting [3]. To mitigate the risks of PWC driving difficulties and limit the risk of accidents, the learning-to-drive process is therefore a key phase. A recent review of the literature [5] highlighted the wide variety of tasks required to learn to drive in a wheelchair and the need for standardized performance assessment. The use of Virtual Reality (VR) simulators could meet these needs in a safe environment and by allowing tasks to be reproduced in a reproducible manner as needed to promote training. Researchers have developed and tested simulators dedicated to wheelchair driving training which can help users adapt their driving strategies to different environment [6–10].

VR-based simulators may be a promising tool for wheelchair driving training allowing more immersive driving situation but needs a good sense of presence i.e. a subjective experience of driving in the environment with the PWC of good quality. Indeed, virtual presence is the ability of being in the virtual world. A high level of presence then allows for greater immersion and therefore for the person to be very close to reality, thus ensuring better training [11, 12]. Indeed, a link between the high level of presence (measured by a 29 items scales) and involvement in the scene has been shown in crisis situations such as a VR situation replicating evacuation in the case of a fire or stress with soldiers [13]. Similarly, this link has also been observed in learning situations with students [14]. Moreover, recent work in the educational sciences has focused on this sense of presence and its link to learning outcomes [15]. One difficulty in using VR-based simulator is the risk of cybersickness. Cybersickness can be considered as a subgroup of motion sickness induced by VR [16], that is stimulated by artificial moving images [17]. Motion sickness is caused by a sensory conflict induced by the disparity in motion between two sensory systems that are the visual and vestibular systems, whereas cybersickness does not involve the vestibular system [18]. Cybersickness is also often experienced during simulation or other VR exposure and must be taken into account in the use of a driving simulator. The symptoms can be nausea, headache, general discomfort, or sweating, and can be experienced during and after exposure to a virtual environment [19]. It seems to be negatively related with sense of presence, which underlines the importance of the level of immersion, to ensure a good level of sense of presence and thus a good quality of learning [12, 20]. Finally, driving difficulties can be experienced by patients with cognitive disorders. They are therefore potential candidates for the use of VR-based simulator as it provides configurable virtual training without physical risks. However, another common difficulty in immersive VR simulation training is the induced mental load [21]. VR-based simulators must therefore be designed so they do not increase too much the mental load in order to enable these patients to benefit from this type of devices.

In this context, the Assistive Devices for empowering People with disabilities through robotic Technologies (ADAPT) project is developing new technologies to facilitate the training of PWCs driving. The aim of this project is to allow a maximum number of users to use a PWC in safety in daily life. For this purpose, a VR-based PWC driving simulator aiming to contribute to driving training for patients has been developed [22]. The simulator provides combined visual feedback and vestibular feedback in order to limit motion sickness. The platform is able to simulate acceleration resulting from user command and centrifugal effects.. The SIMADAPT1 study aims to evaluate the reliability of the proposed simulator compared to the performance on real circuit, in addition to the tolerance of use of the simulator, including assessment of sense of presence, cybersickness and mental load. Even though this study does not deal with the use of the simulator for learning to drive, this is the final objective of this device. We proposed to regular PWC users with neurological disorders to test this simulator prototype on a driving task in a 3D virtual environment replicating 3 real standardized driving circuits [23].

The proposed study involved 3 sessions of driving on circuits of increasing difficulty in VR with the simulator and in R with a standard power wheelchair. Our hypotheses were the following:

- **H1** The simulator enables comparable driving performances between VR and real-life conditions;
- **H2** There is no significant differences regarding the driving ability;
- **H3** There is no significant differences regarding the usability between real and virtual environments;
- **H4** There is no significant differences regarding the mental workload between real and virtual environments;
- **H5** The cybersickness is tolerable;
- **H6** The sense of presence is high in VR condition;
- **H7** Both the cybersickness, presence, and mental workload do not have a significant impact on driving performance between real and virtual conditions.

## Material and methods

### Study design and participants

This study is a prospective, monocentric, randomized and controlled pilot study to investigate the realibility of a PWC simulator. This pilot study was conducted in January 2020 at the rehabilitation center PÃ´le St HÃ©lier in Rennes, France. The study was approved by the People Protection Committee under the ethical approval nÂ°2019-A001306-51.

We screened potential participants 10 days before the beginning of the trial sessions and validated their eligibility for the following inclusion criteria: (1) being over 18 years old; (2) having freely consented to participate in the study; and (3) using a PWC as the main mode of locomotion for more than three months because of a stabilized medical problem. The exclusion criteria were: (1) having difficulties to understand and follow instructions; (2) presenting motor disorders of the upper limb requiring additional driving technical assistance such as specific driving interfaces (head pointer, mouth control, etc.); (3) being pregnant; and (4) unable to express consent. The investigator presented the objectives to each participant as well as the participation modalities according to the validated protocol and delivered an information flier to each participant. The consent form was signed by voluntary participants after a 10 days thinking period.

### Experimental setup

**Fig. 1:**
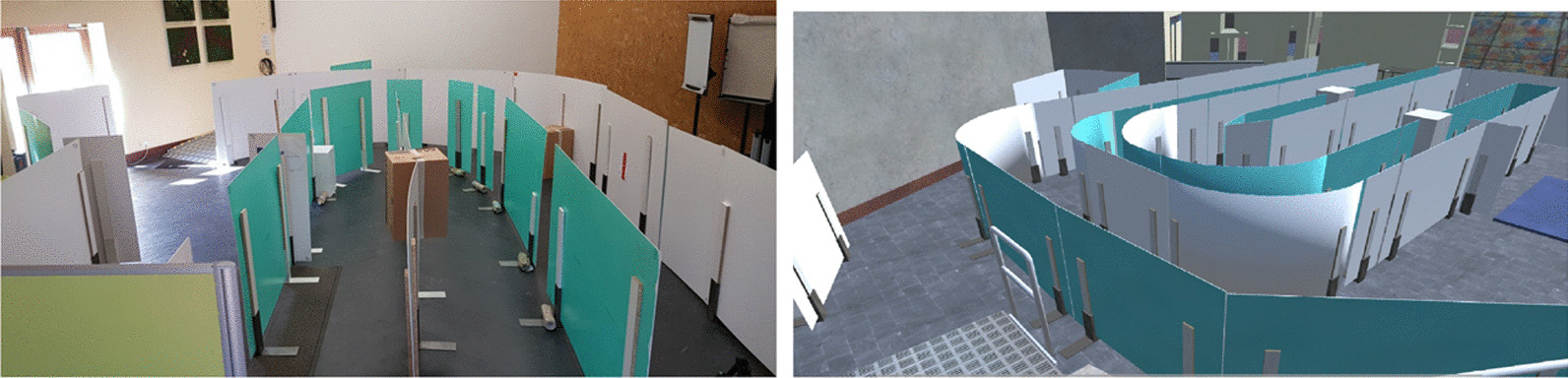
From real to virtual environment: example of virtual scene as a replication of real world scene

A multisensory PWC driving simulator in Virtual Reality was designed within the European Interreg ADAPT project [22][24]. This simulator consists of a four degrees of freedom (pitch, roll, yaw, and heave) mechanical platform designed to induce vestibular feedback [25]. The movements and accelerations of the driving simulator are perceived by the vestibular system of the user while being consistent with the other senses involved in the simulation. Hence, as the vestibular system is responsible for the sense of balance and provides information about the body position, motion sickness is potentially reduced. The user input retrieved from the joystick allows the user to drive the PWC in the virtual environment. A head mounted device displays the virtual environment in which the user navigates. Virtual circuits were modeled in 3D by Unity engine to create test scenarios in VR as illustrated on Fig. 1.

**Fig. 2:**
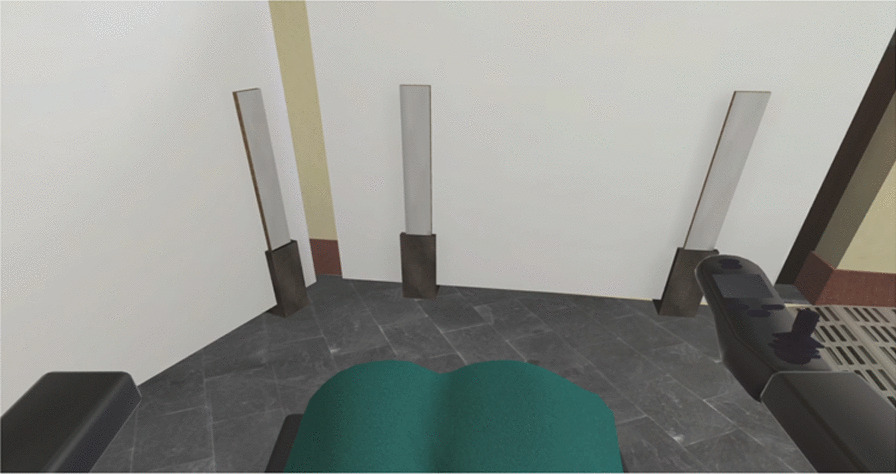
VR simulation capture from user point of view

To ensure that the PWC driving simulator dynamics are the same as real PWC dynamics, the simulator embeds a commercial power module taken from a real PWC. On a real PWC, the power module is responsible for controlling the PWC wheel speeds according to the user joystick input, preset acceleration, and speed parameters. In the simulator, wheel speeds coming from the power module are directly fed into the physics engine responsible for the simulation. Both power modules from real and virtual PWC are set up the same way to provide the same PWC behavior in virtual and real conditions. Speed and acceleration measures, as well as trajectory analysis comparison through motion capture, were performed both in virtual and real conditions to ensure that both wheelchairs were moving at the same speed and had the same behavior. In the virtual environment, the participant visualizes themselves as a first-person avatar without showing the arms and representing the legs covered by a blanket as illustrated on Fig. 2.

**Fig. 3:**
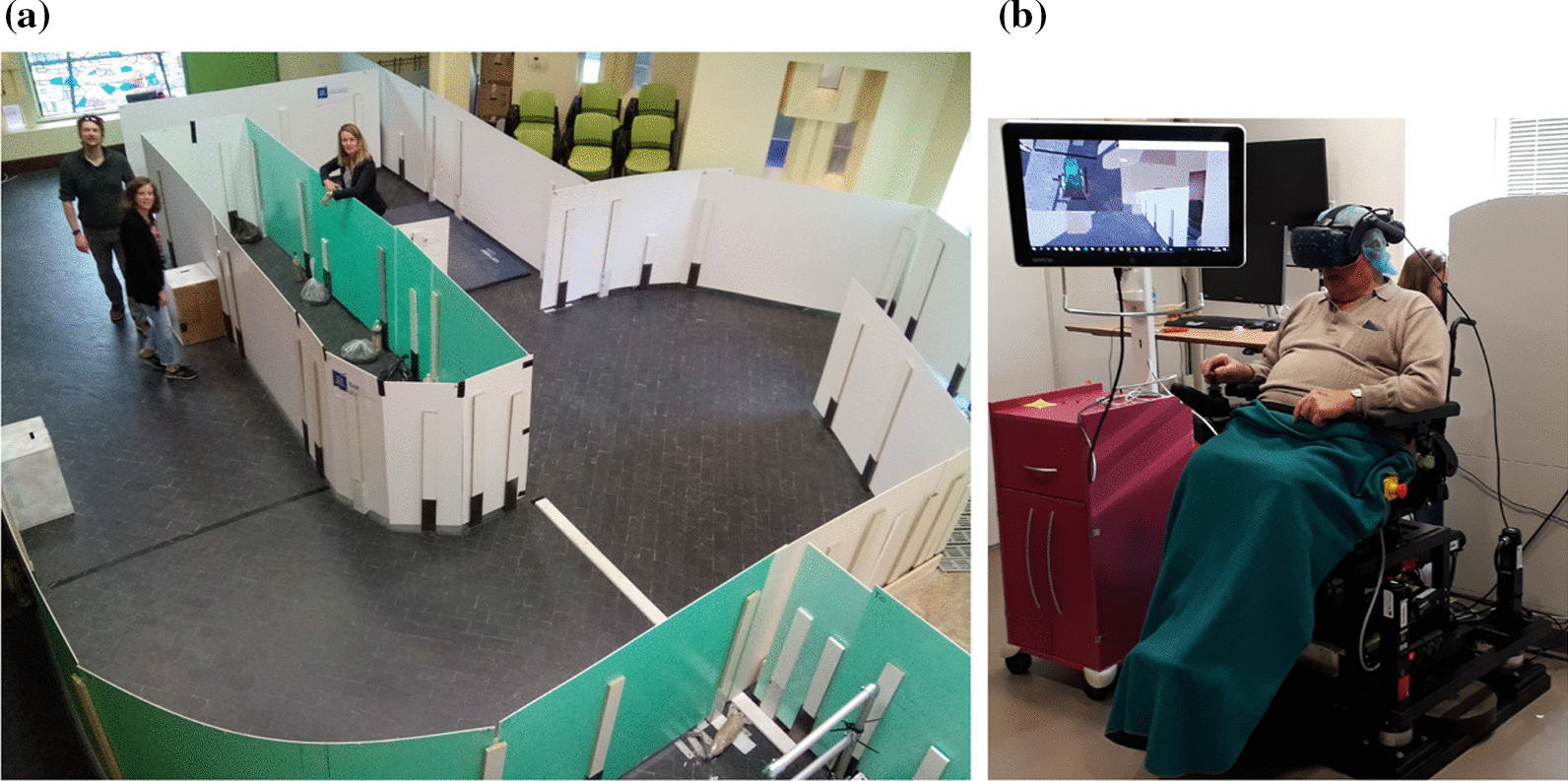
Experimental setup:** a** real circuit and** b** VR-based simulator

The head mounted display used in this trial is a HTC Vive Pro Full kit (certifications: CE 2200, ROHS) that complies with the European R & TTE directives. Standard PWC QUICKIE Salsa M2 from Sunrise Medical (Class I Medical Device CE marked) is used to carry out the trials in R condition. Figure 3 provides an illustration of each experimental setup.

### Experimental procedure

Each participant completed 3 sessions over three weeks in a row. These three sessions corresponded to three progressive levels of driving difficulties. In each session, users tested two conditions: real circuits (R condition) or virtual circuits in the simulator (VR condition) as illustrated on Fig. 3.

Three real circuits intended to assess the driving performances with PWC in indoor situations have been defined according to [23, 26–28]. The first circuit (C1) is composed of several basic tasks such as driving forwards (10 m) and backwards (2 m), turning in place and while moving forwards (90Â°). The second circuit (C2) includes slightly more difficult tasks such as getting through hinged door, ascending and descending a 5^∘^ access ramp, rolling on soft surface (2 m), crossing doorstep and driving through narrow corridors. The third and last circuit (C3) is the most difficult as it includes more difficult maneuvers and tasks such as avoiding a moving obstacle, ascending and descending a 10^∘^ access ramp. In C3, the moving obstacle corresponds to a manual wheelchair passing in front of the participant at a waypoint. In R condition the moving obstacle is pushed by an experimenter whereas it is scripted in VR condition. In both conditions, the moving obstacle is triggered when the wheelchair the patient is using is reaching a specific landmark of the circuit. These different scenarios involve different individual skills as proposed in the Wheelchair Skill Test (WST), which distribution on C1, C2, and C3 is presented in Table 1 [1].

Circuits for the R condition were made of plastic walls, exercise mats, boxes and access ramps. Circuits were replicated to scale in the virtual environment for the VR condition. In addition, vestibular feedback was implemented in the simulator to reproduce the motion and driving behavior of the real PWC used in the R condition.

For each condition, the users completed the circuit twice (T1 and T2 trials). The order between real and virtual conditions was randomized for each session with the Randomizer software (simple randomization). Therefore, each user performed 12 runs, with each run corresponding to a complete lap of the circuit: 2 runs in the R condition and 2 runs in VR condition for each of the 3 sessions.

**Table 1:** Circuits C1, C2, C3 with respect to main items of the WST [1]

Moves controller/tiller away and back	In C1, C2 and C3 circuits
Turns power on and off	
Rolls forwards (10 m)	
Rolls backwards (2 m)	
Turns in place	
Turns while moving forwards ($90^\circ$)	
Gets through hinged door	In C2 and C3 circuits
Ascends $5^\circ$ incline	
Descends $5^\circ$ incline	
Rolls on soft surface (2 m)	
Cross doorstep	
Avoids moving obstacles	Only in C3 circuit
Ascends $10^\circ$ incline	
Descends $10^\circ$ incline	

These circuits are designed to highlight increasing driving difficulties

Before each test session, participants were asked to drive around in the PWC during 5 min in both R and VR conditions to familiarize themselves with the setup. Then, circuits were presented to the participants.

### Study outcomes and measures

The primary outcome is the number of collisions on the three standardized circuits, in R condition versus VR condition. A collision was defined as a contact between the wheelchair and real or virtual parts of the circuits.Two evaluators monitored the participants as they drove around the circuit, and counted collisions during the trial (on a screen for the virtual circuit). An agreement between the two evaluators was necessary to validate a collision. If there was no consensus, the highest number of collisions was retained.

The secondary outcomes consist of the driving speed which was estimated by the time of completion in R and VR conditions.

To assess driving ability, we used the WST 4.2.3 items corresponding to the different courses in R and VR conditions. Each driving skill evaluated through the WST was scored from 0 to 2 (0: fail, 1: pass with difficulty or assistance, 2: pass) by the two occupational therapists involved in the trial. The three circuits were of increasing difficulty and integrated different items of the WST for each one (Table 1).

The mental load under both conditions was measured by the NASA-Task Load Index (NASA-TLX) [29, 30]. The NASA-TLX is a questionnaire in the form of six dimensions which must be scored from 0 to 100. A high score indicates a growing level of intensity. Three dimensions are related to the demands imposed on the subject (mental, physical and temporal) and the three others are related to the interaction of the subject with the task (effort, frustration and performance). A mean score from the six dimensions is computed, with the total obtainable score between 0 and 100. The usability of the PWC under the conditions was evaluated by the Ease of Use Questionnaire (USE) [31]. This questionnaire includes 30-item survey scored on a seven-point Likert rating scales divided in four dimensions exploring usability. These dimensions are namely usefulness, ease of use, ease of learning and satisfaction. For this questionnaire, users were asked to rate agreement with each statement, ranging from strongly disagree (1) to strongly agree (7).

The sense of presence in the virtual environment was evaluated by the Igroup Presence Questionnaire (IPQ) [32]. The IPQ consists of 13 questions and defines the general sense of presence, involvement, spatial presence, and realism. It is composed of three subscales and one additional general item. The general item assessed the general “sense of being there”. The Spatial Presence sub-scale is related to the sense of being physically inside the virtual environment. The involvement subscale aims to evaluate the attention devoted to the virtual environment. The experienced realism sub-scale evaluates the sense of reality attributed to the virtual environment. Each question takes the form of a 7-points scale. Finally, the Simulator Sickness Quantifying (SSQ) measured the feeling of cybersickness and was completed after each VR circuit. The discomfort and cybersickness during the VR condition were also assessed using the Graybiel score [33], which is a questionnaire quantifying the intensity of different symptoms related to cybersickness.

### Data analysis

Data are analyzed using the statistical software R Statistic, version 3.6.1 (2019-07-05). Quantitative analysis of data was expressed as mean and standard deviation and median and inter-quartile range (Md ± IQR). The normality of the data was investigated by the Shapiro test. Data for the 3 circuits in conditions R or VR are not normally distributed. Therefore R and VR conditions were compared using the non-parametric Wilcoxon test. We fitted linear models to study relationship between VR experience parameters (cybersickness, mental load, sense of presence, satisfaction) and driving performances. Post hoc analysis was subsequently conducted to explore any unforeseen correlations or patterns among these variables.

## Results

### Participants

32 users were screened and 31 included: 15 men and 16 women with a mean age of 49.6 ± 13.1 years old. participants were all expert PWC drivers to comply with the objective of this study to validate efficacy and tolerance of the proposed VR-based simulator with people with neurological disorders not experiencing driving difficulties. Characteristics of the participants are presented in Table 2. One user could not perform circuit C2 and two users could not perform circuit C3: this participant refused to continue the experiment due to high level of fatigue.

**Table 2:** Participants’ characteristics: gender, age (years old), diagnosis, and duration of PWC use (in years)

	Gender	Age (years old)	Diagnosis	Duration of PWC use (years)
Patient 01	M	67	Cerebral palsy	2
Patient 02	F	46	Spinal cord injury	1
Patient 03	M	54	Spinal cord injury	4
Patient 04	F	46	Multiple sclerosis	2
Patient 05	F	66	Stroke	6
Patient 06	F	54	Cerebral palsy	9
Patient 07	M	61	Spinal cord injury	1
Patient 08	F	49	Neuro muscular disease	7
Patient 09	M	45	Guillain Barre SD	2
Patient 10	F	27	Cerebral palsy	3
Patient 11	M	65	Multiple sclerosis	2
Patient 12	F	42	Neuro muscular disease	2
Patient 14	M	56	Spinal cord injury	1
Patient 15	M	35	Cerebral palsy	2
Patient 16	M	41	Spinal cord injury	1
Patient 17	F	33	Cerebral palsy	5
Patient 18	F	41	Cerebral palsy	7
Patient 19	F	42	Neuro muscular disease	13
Patient 20	F	64	Multiple sclerosis	6
Patient 21	F	38	Cerebral palsy	9
Patient 22	M	33	Cerebral palsy	3
Patient 23	M	82	Stroke	3
Patient 24	F	41	Cerebral palsy	2
Patient 25	M	34	Spinal cord injury	1
Patient 26	F	41	Stroke	2
Patient 27	M	58	Guillain Barre SD	3
Patient 28	M	42	Spinal cord injury	1
Patient 29	M	67	Spinal cord injury	3
Patient 30	M	44	Multiple sclerosis	8
Patient 31	F	63	Spinal cord injury	3
Patient 32	M	62	Multiple sclerosis	2

### Comparison of driving performances between real and virtual environment

Results are presented in Table 3. There was no significant difference for the number of collisions between the two conditions in C1 and C2. However, a significant difference ($p < 0.01$) can be observed for the number of collisions in the most difficult circuit C3 regarding the two runs: there were more collisions in the VR condition compared to the R condition. Time of completion was also always longer in the VR condition except for the second run in the two last circuits C2 and C3. Therefore, hypothesis H1 is only partially confirmed.

### Driving ability

Concerning the WST score (Table 3), there was no statistically differences between R and VR conditions for the three circuits. H2 is confirmed.

### Usability

Usability measured by the USE questionnaire was high in the two conditions, while being significantly higher for the R condition. Moreover, the most important difference appears for the usefulness dimension, with a score always below 5 in the VR condition. H3 is not confirmed.

**Table 3:** Outcome measures—synthesis

		Circuit 1			Circuit 2			Circuit 3		
		Real Med.	Virtual Med.	Diff. R/V p value	Real Med.	Virtual Med	Diff. R/V p value	Real Med.	Virtual Med.	Diff. R/V p value
Collision	T1	0.00	0.00	NA	0.00	0.00	0.37	0.00	1.00	0.006
	T2	0.00	0.00	0.20	0.00	0.00	0.00	0.00	1.50	0.008
Time(s)	T1	84.00	102.00	<0.001	101.00	103.50	0.01	173.00	187.00	0.001
	T2	84.00	99.00	<0.001	100.00	100.00	0.07	175.00	174.50	0.73
WST		12.00	12.00	0.77	22.00	22.00	0.17	28.00	28.00	0.23
USE	UU	5.75	4.00	<0.001	6.00	4.37	0.007	6.25	5.65	<0.001
	UE	6.40	6.00	0.001	6.30	5.80	<0.001	6.50	6.00	<0.001
	UL	7.00	7.00	0.007	7.00	6.75	0.013	7.00	7.00	0.03
	US	6.33	5.50	0.001	6.00	5.33	0.002	6.33	5.66	0.003
NASA TLX		5.00	18.33	<0.001	6.67	15.00	0.01	10.00	19.17	0.03

For the USE questionnaire, UU corresponds to the “Usefulness” item, UE corresponds to the “Ease of Use” item, UL to the “Ease of Learning” item, US to the “Satisfaction” item, as shown in [31]

### Mental load

The mental load is evaluated through the NASA-TLX. Results are presented in Table 4. It appears that the required mental load is reported to be higher in VR condition when compared to the R condition on all three circuits (C1: $p < 0.001$; C2: $p = 0.01$; C3: $p = 0.03$). Moreover, the mental load does not increase between the C1 and C3 circuits, despite of their increasing difficulty. H4 is also not confirmed.

**Table 4:** Change in Mental Load as assessed by NASA Task Load Index (NASA-TLX) among the three incrementally challenging circuits in Real (R) and Virtual (VR) conditions

	Circuits			p values		
	C1 Median (Mean)	C2 Median (Mean)	C3 Median (Mean)	C1 vs C2	C1 vs C3	C2 vs C3
R	5.00 (7.18)	6.67 (9.38)	10.00 (12.19)	0.44	0.05	0.27
VR	18.33 (21.03)	15.00 (17.81)	19.17 (19.60)	0.39	0.76	0.61

### Cybersickness in VR condition

Cybersickness is evaluated through Graybiel and SSQ scales, which both characterize the cybersickness in VR condition. The related results are presented in Table 5. Regarding the SSQ scores (total possible score up to 48), participants rated their cybersickness to be relatively low (mean SSQ values are equal to 4.25, 3.43 and 4.72 with respect to C1, C2 and C3 circuits). For the Graybiel, the highest possible score is 48 while a score above 16 is corresponding to frank sickness (level 4), and a score under 3 is considered as a slight discomfort (level 1). In our results, the maximum Graybiel score of 2.07 is reached on the circuit C3. Sickness was higher in the most difficult circuit (C3). If there was a significant decrease on the Graybiel score for C2 regarding the score for C1, it is different concerning the SSQ. The Graybiel scores were not statistically different between C1 and C3 ($p = 0.48$).Therefore, H5 is not confirmed.

**Table 5:** Outcome measures related to cybersickness for the three circuits

	Circuits			p values		
	C1 Median (Mean)	C2 Median (Mean)	C3 Median (Mean)	C1 vs C2	C1 vs C3	C2 vs C3
Graybiel	1.00 (1.83)	1.00 (0.82)	1.00 (2.07)	0.014	0.48	0.018
SSQ	3.00 (4.25)	1.50 (3.43)	2.00 (4.71)	0.15	0.70	0.09

Graybiel and Simulator Sickness Quantifying (SSQ) tests were used

### Sense of presence

**Fig. 4:**
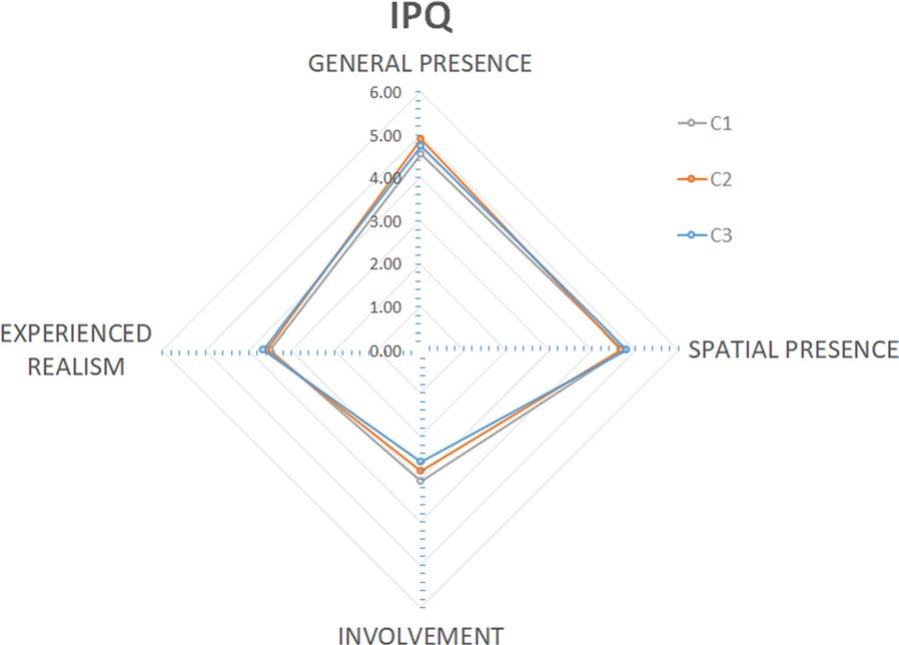
Graphical representation of IPQ results. The IPQ is composed of three subscales and one additional general item. The general presence item assessed the general “sense of being there”

As shown in Fig. 4, the Igroup Presence Questionnaire (IPQ) results showed an acceptable general and spatial presence with the virtual environment regarding to [34]. Results were similar for the 3 circuits, with lower scores for experienced realism and involvement. H6 is confirmed.

### Relationship between driving performances and VR experience

**Fig. 5:**
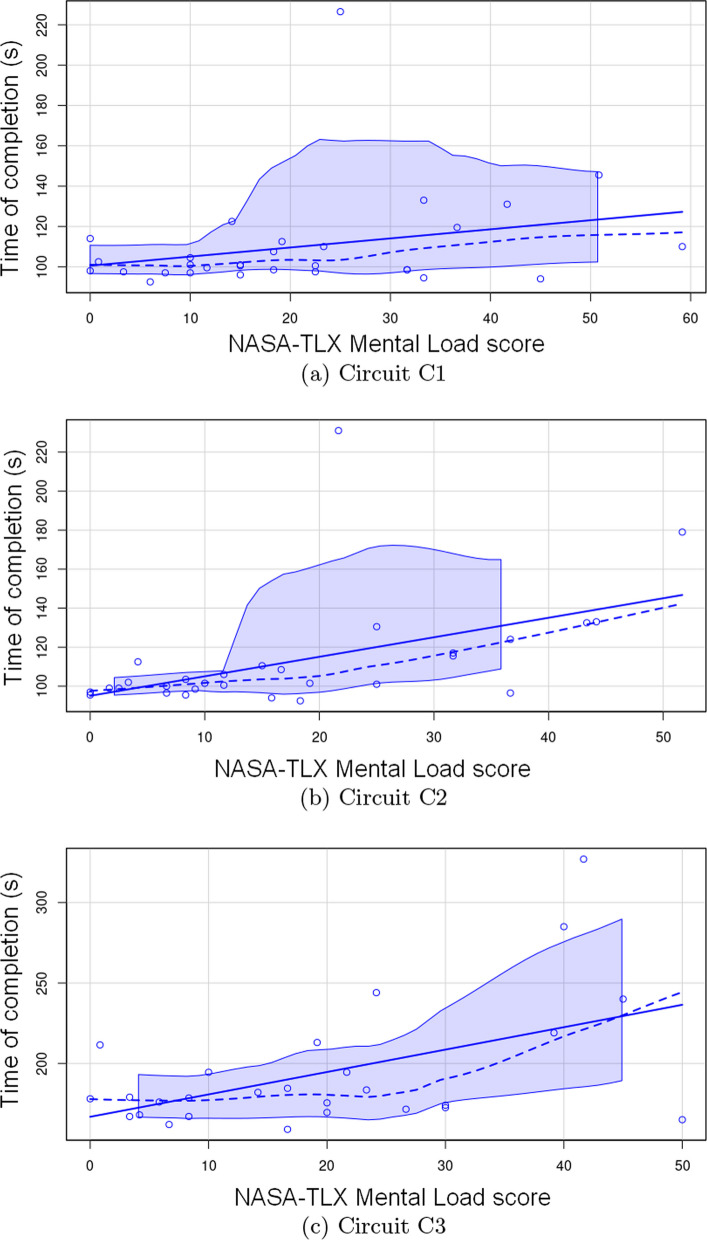
Relationship between mental load and time of completion across three circuits; scatter plots with dashed line: LOESS (LOcally Estimated Scatterplot Smoothing) and confidence intervals; Solid line: Fitted regression line

To study relationships between time of completion and the VR experience measures, a simple linear regression has been fitted to the data (Fig. 5) in order to evaluate the relationship between time of completion and mental load (NASA-TLX workload score), satisfaction of use (USE score), Cybersickness (Graybiel and SSQ scores) and sense of presence (IPQ [35]).

As the circuits are of increasing complexity, the analysis was carried out for each circuit individually, to take these differences into account. For circuit C1, the interaction between time of completion and these five variables have been found to be statistically not significant ($R2 = 0.17$, $p = 0.747$, $adj.R2 = -0.11$). Nevertheless, we observe an effect of the NASA-TLX based variable which has been found to be statistically significant and positive ($\beta = 1.43$, $95\%$ CI [0.09, 2.77], $p = 0.037$; $Std.\beta = 0.72$, $95\% CI [0.05, 1.39]$). For C2, the same behavior is observed: there is no significant relationship between time and VR experience variables, except for the NASA-TLX based variable, which appears to be statistically significant and positive ($\beta = 1.43$, $95\%$ CI [0.09, 2.77], $p = 0.037$; $Std.\beta =0.72$, $95\%$ CI [0.05, 1.39]). For C3, the linear prediction model is not statistically significant ($R2 = 0.47$, $p = 0.079$, adj.$R2 = 0.26$), and the NASA-TLX based variable has a statistically significant and positive effect ($\beta = 1.71$, $95\%$ CI [0.21, 3.21], $p = 0.028$; Std.$\beta =0.63$, $95\%$ CI [0.08, 1.18]).

We performed the same analysis for the number of collisions in VR condition. For all the circuits, each variable has a statistically non significant effect. Only the effect of NASA-TLX score is statistically significant and positive ($\beta = 0.08$, $95\%$ CI $[2.90e-05, 0.16]$, $t(18) = 2.10$, $p = 0.050$; Std.$\beta = 0.57$, 95$\%$ CI $[2.11e-04, 1.14]$). Regarding NASA-TLX based variable, H7 is only partially confirmed.

## Discussion

### Results and discussion

In this study, the comparison between driving performances in VR and R conditions show no statistically difference in easy to moderate difficulty circuits (C1 and C2), both for the number of collisions and the time to completion. On these circuits, only a few collisions were reported in the two conditions which is probably due to the expertise of the participants with respect to the driving of a PWC. The circuit C3 was the most difficult circuit with a statistically significant higher number of collisions in the VR condition. Conversely the time of completion of C3 remained significantly higher in R condition with respect to VR condition for the first round, but not for second round. This could be explained by the mental load, but also by the feeling of safety induced by virtual reality, leading people to take more risks by driving faster. Collisions and time of completion are classically the main assessment criteria during a driving test on standardized circuits [23, 36, 37]. Moreover, the comparison between real and virtual conditions for the task of driving a wheelchair has already been studied, and showed similar results. Indeed, Cooper et al. [38] compared wheelchair driving tasks in virtual and real conditions with two types of joysticks. They demonstrated that there were just few differences in task completions and that the driving in a virtual environment relates to driving in a real environment. Moreover, Archambault et al. [39] also highlighted that the driving performances in a wheelchair simulator were not statistically different between virtual and real environments, although task completion was higher in the simulator for the most difficult task.

Frederiksen et al. [21] also showed that the mental load is increased in VR and hypothesized that this can be due to the fact that VR can consist of more virtual elements (visual stimulation, dynamical virtual objects, etc.) and interactions. This is however not the case in our study as we replicate the real environment strictly in the virtual environment, with no additional objects or visual stimulation. In the other hand, the use of a Head Mounted Display (HMD) device can have an impact on the mental load. Winter et al. [40] also observed that the mental load was significantly higher in immersive condition compared with training without VR. In our experience, we have similar results with an increase in mental load in all the circuits. However, despite an increase in complexity, there are no significant difference in mental load between the 3 circuits. Therefore, it can be assumed that the mental load increase is not sufficient to impair wheelchair driving in circuits C1 and C2. For the most difficult circuit C3, this does not seem to be the case either, despite a higher number of collisions. Nevertheless, we have to take into account that the order of the C1, C2 and C3 was not randomized, there might have been a learning effect for the last round, which could explain the lack of statistical significance. Moreover, performance also seems to be linked to cognitive load, with perhaps a threshold effect. The second hypothesis is that the perception of the risk of collisions in VR is different because there is no possibility of injury in the virtual environment, thus encouraging greater risk-taking by users. We then expect that we could increase the risk perception by enhancing the quality of the virtual environment. In our study, the sense of presence is acceptable and leads to a good immersion in virtual environment, but the realism is reported as rather weak [41]. Gotzelmann et al. [42] observed that adding virtual pedestrians can allow driving in a urban environment conditions, with interactions between the virtual pedestrians and the wheelchair driver in the simulator. At this stage, and for this study, the proposed simulator does not incorporate virtual pedestrians to interact with. Such a feature would eventually increase the realism of our simulator, with an impact on the sense of presence as it adds realness to the virtual content [34]. The sense of presence generally depends also on the type of display, the orientation of the visual field and the visualization of the user avatar. In our study, the avatar is restricted to a blanket covering the legs. This has been done to avoid user-avatar visual similarity mismatch issues which can impact the behavior of participants. We could explore avatar more advanced design to provide more realistic PWC driving in our virtual environment. In addition, we can hypothesize that an improvement of the virtual environment could decrease the mental load. Indeed, Kamaraj et al. showed the importance of the quality of both virtual environment and sense of presence regarding their impact on the mental load [43]. In this study, users reported an acceptable general and spatial presence, but the virtual environment appears insufficient in terms of realism. Improvements are needed in this field for future development.

Contrary to previous studies [19, 44], we observed an acceptable low level of cybersickness, although it could be decreased with further technical improvements. For example, Winter [40] synchronized speed of visual motion in the virtual environment with the physical speed of a treadmill with good results. The technological improvement of such a simulator by adding innovative advanced visual and vestibular perception modalities regarding the PWC motion and dynamics seems to be efficient to reduce motion sickness. Moreover it seems also important to gradually and regularly expose users to immersive VR experiences [45]. In our experimental procedure, each run only lasts a short period of time. The next experimental step will consist of longer immersive and repeated sessions in order to enhance the user virtual experience and to assess learning effects.

### Perspectives

The place of the simulators in the rehabilitation process remains to be determined. Indeed, 25$\%$ of wheelchair accidents are caused by PWC users that represent $10\%$ of the wheelchair users [4, 46, 47]. In addition, 100 000 wheelchair accidents were recorded in US in 2006, twice as many as in 1991 [48]. This shows that the use of power mobility assistance devices comports risks. In particular, falling and tipping are the most common recorded accidents but with PWC, the most frequent reported accidents are due to direct collisions. Ummat et al. noticed that 7.6$\%$ of accidents cause injuries to the human environment i.e. people around the wheelchair, and not directly the PWC driver themselves [49, 50]. This shows that the risk is important, and it can be easily understandable that the lack of security sometimes leads users to restrict their use of the wheelchair. This highlights the importance of training regarding PWC driving. Currently, real-life assessment remains the Gold Standard. In the present study users appreciated the simulator, but their satisfaction was greater in real situations than in virtual situations on the USE scale over all domains, even if score remained high in VR conditions. Archambault et al. also noticed that participants were neutral in terms of wanting to continue using the simulator particularly when their performance was good [39]. Hence, simulator may be a promising approach to complement training received in rehabilitation centers, especially in case of learning difficulties. Different scenarios can be imagined to optimize the driving control in total safety. Though, simulators remain an expensive technology which is not accessible to everyone. In the first instance, the ecological approach should be preferred with the possible use of a circuit. Note that our VR-based simulator is currently only at the prototype stage and our study has only involved simulations on driving circuits. The creation of more complex environments is necessary in order to consider simulations of everyday life.

## Conclusion

In this paper, we evaluated a power wheelchair driving simulator in virtual reality through a pilot study involving 31 expert power wheelchair users. Driving performance were not statistically different when the same circuit was run with the simulator and in real-life for circuits for C1 et C2. Although results were statistically different for C3 regarding the number of collisions which was higher in VR, there were no significant adverse effects such as cybersickness. Virtual reality may not be totally comparable to a real-life situation, but it is nevertheless a effective tool for putting users in difficulty into safe driving situations. Our study thus demonstrates that the proposed simulator is suitable for driving task scenarios in simulation, and show the value of the simulator for driving training applications. Still, mental load remains high during virtual immersion and raises the question of the use of VR for individuals with cognitive disorders who may be in difficulty in complex situations. If the use of simulator may probably not be proposed to these individuals as a first step in driving training, it clearly can be a useful tool for patients unable to safely drive a PWC in order to help them to acquire the necessary driving skills in safe, adaptable, and repeatable conditions. Further studies should be conducted to assess the quality of skill transfer for novice drivers from the simulator to the real world.

## Data Availability

Datasets used and analyzed during the current study are available from the corresponding author on reasonable request.

## Abbreviations

VR: Virtual reality
PWC: Power wheelchair
TBI: Traumatic brain injury
CP: Cerebal palsy
WST: Wheelchair skill test
NASA-TLX: NASA Task Load questionnaire
USE: Ease of Use Questionnaire
IPQ: Igroup Presence Questionnaire
SSQ: Simulator Sickness Questionnaire
C1: Circuit 1
C2: Circuit 2
C3: Circuit 3
T1: Trial 1
T2: Trial 2
